# Phenotypic detection of methicillin resistance, biofilm production, and inducible clindamycin resistance in *Staphylococcus aureus* clinical isolates in Kathmandu, Nepal

**DOI:** 10.1186/s41182-022-00460-1

**Published:** 2022-09-21

**Authors:** Sujina Maharjan, Mehraj Ansari, Pawan Maharjan, Kul Raj Rai, K. C. Sabina, Hari Prasad Kattel, Ganesh Rai, Shiba Kumar Rai

**Affiliations:** 1 Department of Microbiology, Shi-Gan International College of Science and Technology, Kathmandu, Nepal; 2 Phect-Nepal Model Hospital School of Medical Laboratory Sciences, Kathmandu, Nepal; 3 Key Laboratory of Fujian-Taiwan Animal Pathogen Biology, College of Animal Science, Fujian Agriculture and Forestry University, Fuzhou, China; 4 Department of Microbiology, Tribhuvan University Teaching Hospital, Kathmandu, Nepal; 5 Department of Microbiology, Nepal Medical College and Teaching Hospital, Kathmandu, Nepal

**Keywords:** Biofilm, Clindamycin, MRSA, MSSA, Multidrug resistance, Vancomycin

## Abstract

**Introduction:**

Methicillin resistance, inducible clindamycin resistance (ICR), biofilm production, and increased minimum inhibitory concentration (MIC) of vancomycin in *Staphylococcus aureus* are major causes of antibiotic treatment failure and increased morbidity and mortality. The surveillance of such isolates and the study of their antimicrobial pattern are essential in managing the infections caused by these isolates. This study aimed to determine methicillin resistance, biofilm production, and ICR in *S. aureus* isolates from a tertiary care hospital in Kathmandu, Nepal.

**Materials and methods:**

A total of 217 *S. aureus* isolated from different samples were processed following standard laboratory procedures. Antibiotic susceptibility testing was performed by the Kirby–Bauer disk diffusion technique. Methicillin-resistant *S. aureus* (MRSA) were identified by the cefoxitin disk diffusion test, and biofilm producers were examined using the microtiter plate technique. D-test and E-test were performed to determine inducible clindamycin resistance and minimum inhibitory concentration of vancomycin, respectively.

**Results:**

Among the 217 *S. aureus* isolates, 78.3% were multidrug-resistant (MDR), 47.0% were MRSA, 62.2% were biofilm producers, and 50.7% showed ICR. All MRSA isolates exhibited MIC levels of vancomycin within the susceptible range. Biofilm producers and MRSA isolates showed elevated antimicrobial resistance. MRSA was significantly associated with MDR. Biofilm-producing and multidrug-resistant MRSA isolates showed significantly higher MIC levels of vancomycin (*p* = 0.0013 and < 0.0001, respectively), while ICR was significantly higher in MDR (*p* = 0.0001) isolates.

**Conclusion:**

High multidrug resistance, MRSA, and ICR in this study call for routine evaluation of antibiotic susceptibility patterns of *S. aureus*. Vancomycin can be used to treat serious staphylococcal infections. Clindamycin should be prescribed only after performing the D-test. Drugs like teicoplanin, chloramphenicol, doxycycline, amikacin, and levofloxacin can treat MRSA infections.

**Supplementary Information:**

The online version contains supplementary material available at 10.1186/s41182-022-00460-1.

## Introduction

*Staphylococcus aureus* is a major opportunistic human pathogen causing many clinical infections. The rates of infections caused by staphylococci, both community, and hospital-acquired strains, are increasing steadily [1]. Antimicrobial therapy is widely practiced for the management of staphylococcal infections. Broad-spectrum antibiotics, including conventional penicillin, aminopenicillins, cephalosporins, and other β-lactam antibiotics are the mainstay of antibacterial therapy [2]. However, the emerging resistance in *S. aureus* has left us with very few therapeutic alternatives to treat the infections caused by them [1].

One of the most common causes of drug resistance in *S. aureus* is the evolution of methicillin-resistant *S. aureus* (MRSA) [3, 4]. MRSA usually shows a multidrug-resistant pattern, resistance to penicillin and other antimicrobial classes including macrolides, fluoroquinolones, aminoglycosides, tetracyclines, and lincosamides. Therefore, MRSA often causes antibiotic treatment failure, increased morbidity, and mortality and has been a major cause of severe hospital and community-acquired infections [3]. Biofilm formation is another important drug resistance mechanism exhibited by *S. aureus* and is one of the biggest challenges in antimicrobial therapy. Biofilm producers exhibit elevated resistance to all classes of antimicrobial agents and resist clearance by the host defense system leading to persistent and recurrent device-related infections [5]. Furthermore, once a biofilm is formed, antibiotics fail to penetrate the dense matrix and cannot target bacterial cells in the deeper layers of the biofilm, which complicates treatment [5].

Vancomycin has been the mainstay of the first-line therapy for severe MRSA infections [6]. However, the irrational use of vancomycin has led to a higher likelihood of mortality or treatment failure among patients infected with MRSA [6]. Since the first report in 1997 [7], cases of vancomycin resistance-related treatment failures have been increasingly reported worldwide. Furthermore, the emergence of high‐level vancomycin-resistant *S. aureus* (VRSA) has been reported since 2002 [8, 9]. Bacterial biofilm growth is inversely related to vancomycin concentration in the biofilm, and even very high drug concentrations of vancomycin cannot confer efficient killing effects to bacteria embedded in the biofilm [10]. Clindamycin, a macrolide–lincosamide streptogramin B (MLS_B_) family of antibiotics, is another reserve drug usually advocated for treating Staphylococcal infection [11]. However, the widespread use of drugs has led to the emergence of many resistant isolates to clindamycin. Resistance to MLS_B_ arises mainly by an active efflux mechanism coded by the *msr*A gene or erm genes. The genes encode enzymes capable of conferring inducible (iMLS_B_) or constitutive (cMLS_B_) resistance to all three groups of drugs via methylation of the 23S rRNA [12]. Constitutive resistance can be detected by the routine disk diffusion method, but it fails to detect inducible resistance, which appears sensitive to clindamycin on routine testing, leading to inappropriate clindamycin therapy, resulting in treatment failure [11]. Hence, it is essential to identify the iMLS_B_ isolates before clindamycin therapy. Such isolates can be effectively identified by the double disk approximation test (D-test), a simple and CLSI-recommended phenotypic test that can separate strains with genetic potential (i.e., the presence of erm genes) to become resistant during therapy from strains that are fully susceptible to clindamycin [13].

In a nutshell, the surveillance of antimicrobial patterns of staphylococcal isolates is essential in understanding new and emerging resistant patterns and the management of the hospital and community-acquired infections [14]. Although numerous reports on MRSA have been published, there are only a few studies reporting biofilm production and ICR in *S. aureus* from Nepal. This study aimed to phenotypically determine methicillin resistance, biofilm production, and ICR in *S. aureus* isolates from a tertiary care hospital in Kathmandu, Nepal.

## Materials and methods

### Study design

A laboratory-based cross-sectional study was conducted from October 2018 to March 2019 at the Tribhuvan University Teaching Hospital (TUTH), a tertiary care center in Kathmandu, Nepal. The study population included patients of all age groups and sex visiting the inpatient and outpatient departments of the hospital to whom culture tests had been referred.

### Specimen collection and processing

A total of 217 *S. aureus* were isolated from different types of samples; pus (*n* = 194), sputum (*n* = 6), blood (*n* = 4), body fluids (*n* = 9), and urine (*n* = 4), received in the bacteriology lab of TUTH, using standard microbiological techniques. Briefly, the samples were inoculated in blood agar (BA) and mannitol salt agar (MSA) plates (HiMedia Pvt. Ltd, India). The isolates that were creamy white colonies showing hemolysis in the BA and golden yellow colonies in the MSA plates were further identified as *S. aureus* based on Gram stain, urease, Voges Proskauer (VP), catalase, DNase and coagulase tests [15, 16]. (Additional file 1: Photograph S1A and B).

### Antibiotic susceptibility testing (AST)

The antibiotic sensitivity test of *S. aureus* was performed by the modified Kirby–Bauer method as recommended by CLSI [17] on Mueller–Hinton agar (MHA) (HiMedia Pvt. Ltd., India). The antibiotic disks (HiMedia Pvt. Ltd., India) used were amikacin (30 µg), amoxycillin (20 µg), amoxyclav (30 µg), cefixime (5 µg), cefoxitin (30 µg), cephalexin (30 µg), chloramphenicol (30 µg), ciprofloxacin (5 µg), clindamycin (2 µg), cloxacillin (1 µg), cotrimoxazole (25 µg), doxycycline (30 µg), erythromycin (15 µg), imipenem (10 µg), levofloxacin (5 µg), meropenem (10 µg), oxacillin (1 µg), teicoplanin (30 µg), and vancomycin (30 µg). The interpretation of the results was made as described in CLSI M100-S24 [17]. Isolates resistant to at least one agent in three of more antimicrobial classes were considered multidrug-resistant (MDR) [18] (Additional file 1: Photograph S1C).

### Detection of MRSA

MRSA detection was performed by the cefoxitin disk diffusion test [17]. The interpretive criteria used were: zone size ≥ 22 mm: susceptible (MSSA), zone size ≤ 21 mm: resistant (MRSA).

### Detection of biofilm producers

Biofilm was detected by the microtiter plate technique [19]. A 10 µl of bacterial suspension adjusted to 0.5 McFarland standard in normal saline was added to 1 ml brain heart infusion (BHI) broth with 1% glucose. A 200 µl of the diluted bacterial suspension was passed on the microtiter plate and incubated in static condition for 18 h at 35 ± 2 °C. Triplets were run for each isolate for biofilm formation. After incubation, the microtiter plates were vigorously washed with phosphate buffer (pH = 7.2) three times to remove all planktonic bacteria and non-biofilm adhesion of bacteria. Then, each well was stained using 200 μl of 0.1% safranin for 5 min and washed with distilled water. Then, the plates were blotted on tissue paper for 30 min. Finally, 200 μl of 30% acetic acid was added to each well to dissolve the safranin. The optical density (OD) was measured in a semiautomatic ELISA reader (Thermo-Fisher Scientific, USA) with a primary filter of 490 nm and a secondary filter of 640 nm. For quantification of the biofilm of each isolate, the average OD of the control was calculated from three negative controls, and the average OD of the test was calculated from the triplicates of each isolate. The result was interpreted as follows: OD ≤ ODc: non-adherent, ODc < OD ≤ 2 × ODc: weakly adherent, 2 × ODc < OD ≤ 4 × ODc: moderately adherent, and 4 × ODc < OD: strongly adherent. (Additional file 1: Photograph S1E).

### Detection of inducible clindamycin resistance (ICR)

The test for inducible clindamycin resistance was performed by the D-test [20]. The isolates showing erythromycin resistance were lawn cultured over the MHA plate (HiMedia Pvt. Ltd., India). Erythromycin (15 µg) and clindamycin (2 µg) disks (HiMedia Pvt. Ltd., India) were placed at a 15-mm edge-to-edge distance. After overnight incubation at 37 °C, different phenotypes were interpreted as follows: (Additional file 1: Photograph S1D).

i. Moderate-sensitive (MS) phenotype: resistant to erythromycin (zone size ≤ 13 mm), but sensitive to clindamycin (zone size ≥ 21 mm) and giving a circular zone of inhibition around clindamycin (D-test negative).
ii. Constitutive MLS_B_ (cMLS_B_) phenotype: resistant to both erythromycin (zone size ≤ 13 mm) and clindamycin (zone size ≤ 14 mm) with the circular shape of the zone of inhibition around clindamycin.
iii. Inducible MLS_B_ (iMLS_B_) phenotype: resistant to erythromycin (zone size ≤ 13 mm), but sensitive to clindamycin (zone size ≥ 21 mm) and giving a D-shaped zone of inhibition around clindamycin with flattening toward erythromycin (D-test positive).

### MIC of vancomycin

MIC of vancomycin was detected by the epsilometer test (E-test). Commercially available E (EZY MIC™, HiMedia Pvt. Ltd) was placed over previously dried MHA plates seeded with the test strain. The plate was incubated at 37 °C for 18–24 h under aerobic conditions. MIC results were interpreted according to the CLSI guidelines and manufacturer’s protocol: MIC < 2 μg/ml: sensitive, MIC 4–8 μg/ml: intermediate, and MIC > 16 μg/ml: resistant [17] (Additional file 1: Photograph S1F).

### Quality control

The tests were performed adopting standard microbiological techniques [15] following CLSI guidelines. A purity plate was used for quality control of biochemical tests. Similarly, S. *aureus* (ATCC 25923) was used as a positive control along with appropriate negative control in each step to standardize the tests.

### Data analysis

Primary data were entered in Microsoft Excel 2016. Later, GraphPad Prism was used for the figures and statistical analysis. Chi-square test was used to find the association between two variables. Similarly, an unpaired *t*-test with Welch’s correction (Welch’s test) was used to determine the significance of the difference in the MIC values of different MRSA isolates. A *p*-value of < 0.05 was considered statistically significant.

## Results

Of the 217 growth positive samples, 52.5% were from females and 47.5% from the males. The isolation of *S. aureus* was higher in the age group 0–30 years than in the elderly age groups (Table 1).

**Table 1:** Sex and age group-wise distribution of *S. aureus* isolates

Age group (years)	Sex		Total
	Male (*N*)	Female (*N*)	
0–10	35	19	54
11–20	25	26	51
21–30	17	38	55
31–40	12	12	24
41–50	5	5	10
51–60	4	8	12
60+	5	6	11
Total	103 (47.5%)	114 (52.5%)	217

**Fig. 1:**
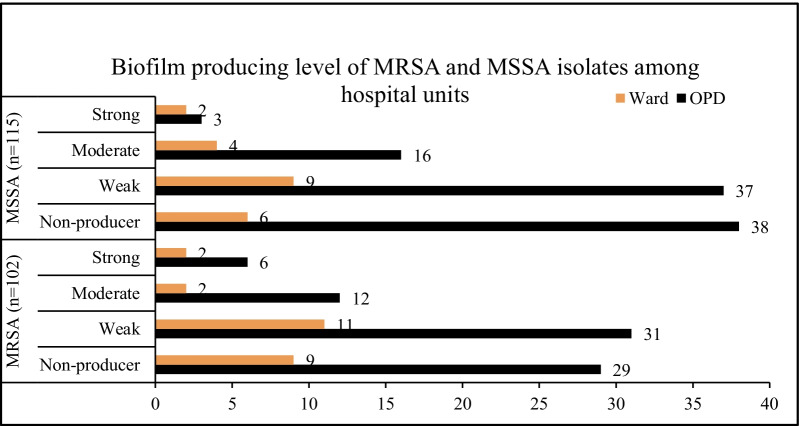
Biofilm production level of MRSA and MSSA isolates among hospital units

Among the 217 *S. aureus* isolates, 78.3% (*n* = 170) were MDR. Similarly, 53.0% (*n* = 115) and 47.0% (*n* = 102) isolates were characterized as MSSA and MRSA, respectively. Of the 102 MRSA isolates, 98 (96.1%) were MDR, and there was a strong association between MDR and MRSA (*p* < 0.0001). Biofilm production was observed in 62.2% (*n* = 135) isolates: 40.6% (*n* = 88) weak, 15.7% (*n* = 34) moderate and 6.0% (*n* = 13) strong producers. There was no significant association of biofilm production with multidrug resistance (*p* = 0.935) and methicillin sensitivity (*p* = 0.879). Most biofilm producers (48.4%, *n* = 105) were from OPD (Table 2 and Fig. 1).

**Table 2:** Association of biofilm production with multidrug resistance and methicillin sensitivity

	Biofilm		Total	*p*-value
	Producer	Non-producer		
Multidrug resistance				
MDR	106 (62.4%)	64 (37.6%)	170 (78.3%)	0.935
Non-MDR	29 (61.7%)	18 (38.3%)	47 (21.7%)	
Methicillin sensitivity				
MRSA	64 (62.7%)	38 (37.3%)	102 (47.0%)	0.879
MSSA	71 (61.7%)	44 (38.3%)	115 (53.0%)	
Total	135 (62.2%)	82 (37.8%)	217 (100%)	

MDR: multidrug-resistant, MRSA: methicillin-resistant *S. aureus*, MSSA: methicillin-sensitive *S. aureus*

### Antibiotic sensitivity pattern of *S. aureus*

**Fig. 2:**
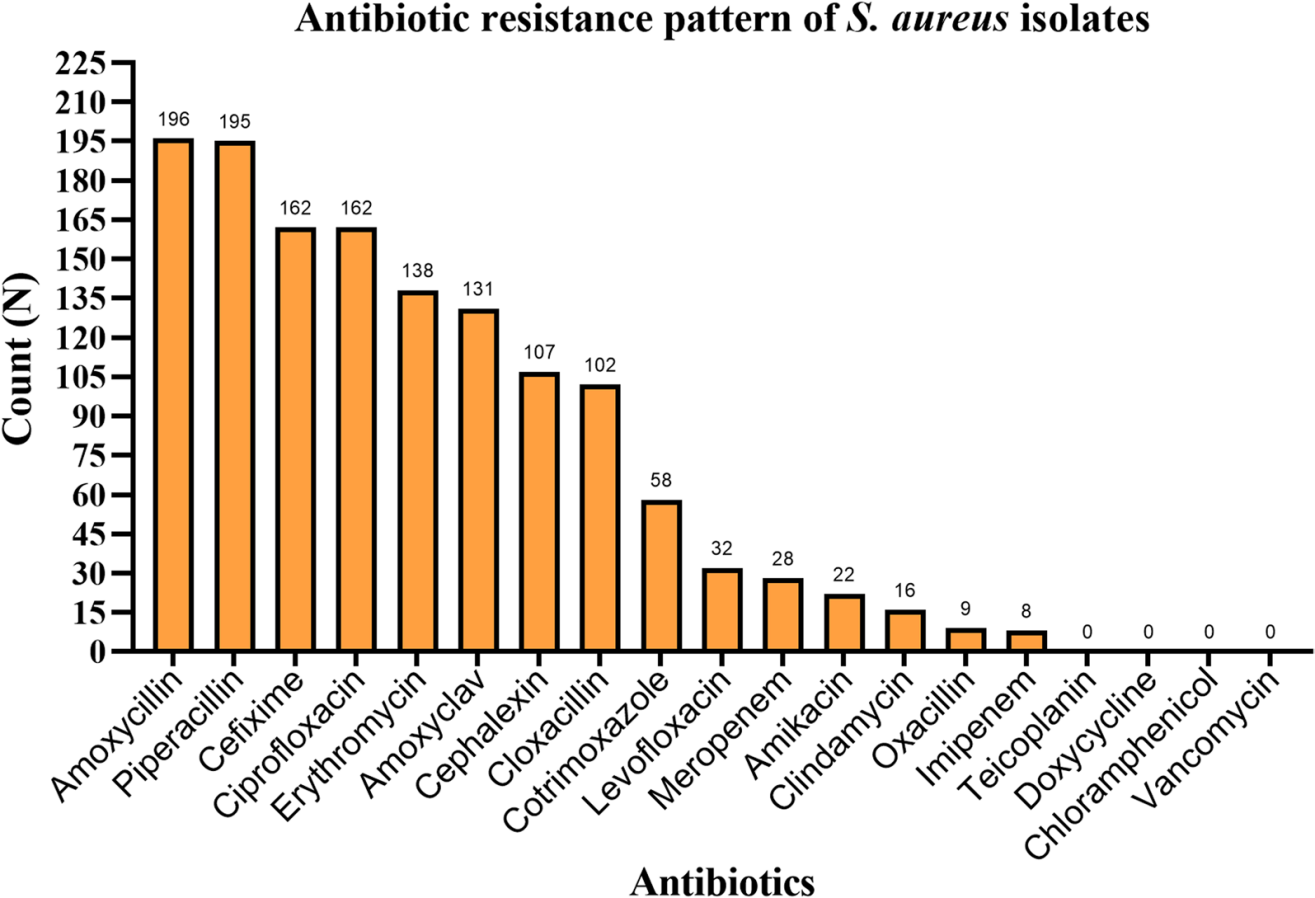
Antibiotic resistance pattern of *S. aureus* isolates

**Fig. 3:**
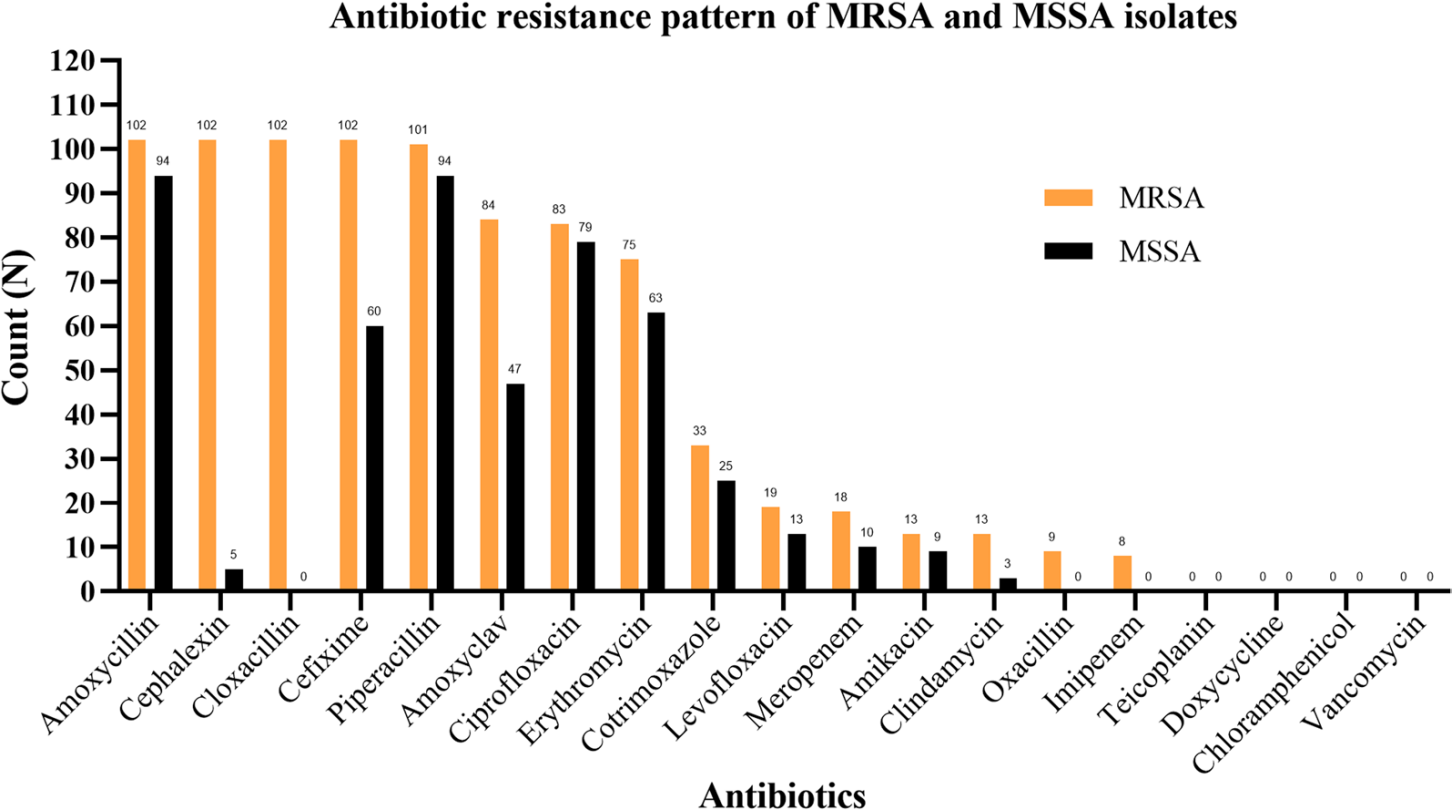
Antibiotic resistance pattern of MRSA and MSSA isolates

**Fig. 4:**
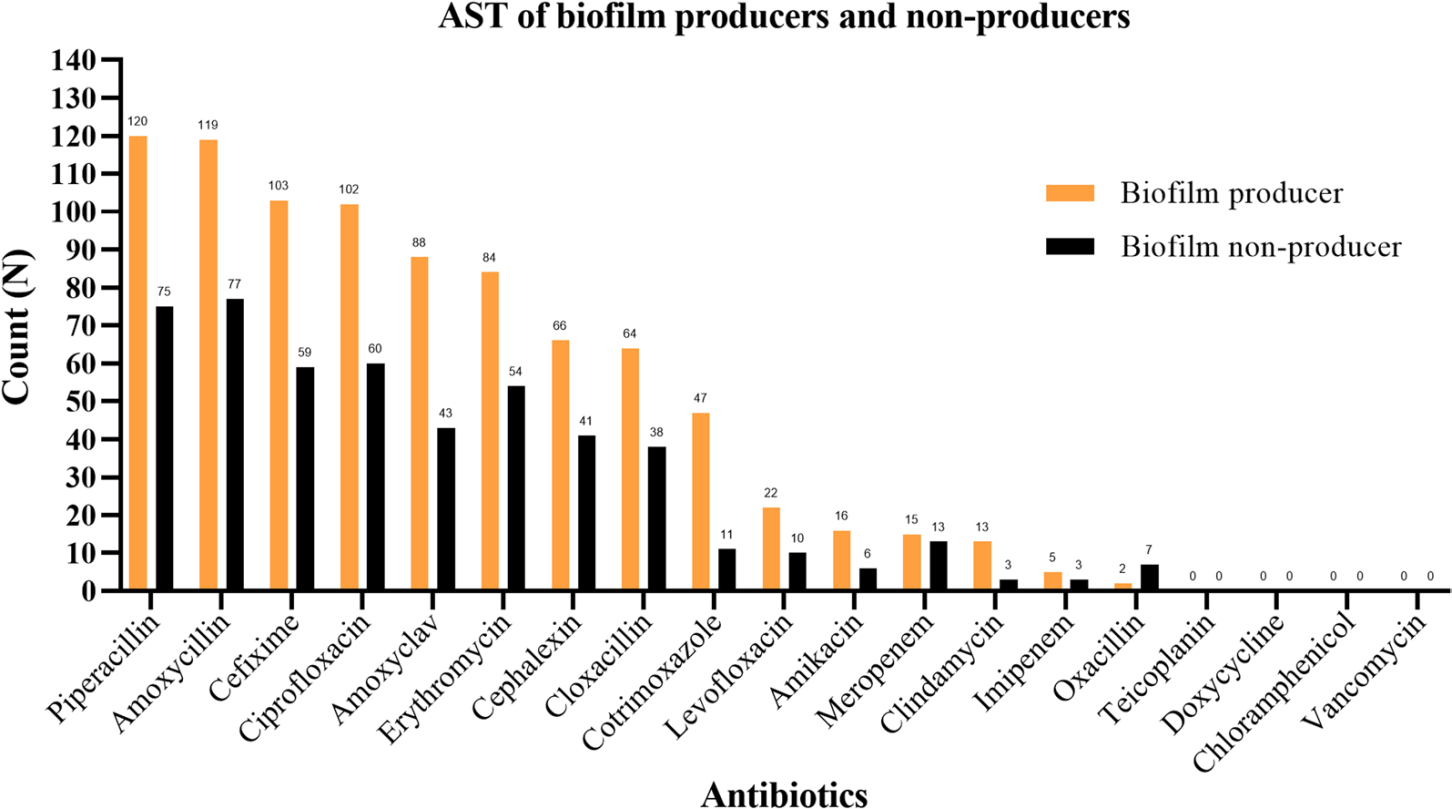
Antibiotic resistance pattern of biofilm producers and non-producers

MRSA isolates were highly resistant to amoxycillin, cephalexin, cefixime, cloxacillin, and piperacillin. All the *S. aureus* isolates were susceptible to vancomycin, teicoplanin, doxycycline, and chloramphenicol. Except for the four antibiotics, MRSA isolates and biofilm producers had elevated resistance compared to MSSA and biofilm non-producers, respectively, to all other antibiotics (Figs. 2, 3, and 4).

### Inducible clindamycin resistance in *S. aureus*

**Fig. 5:**
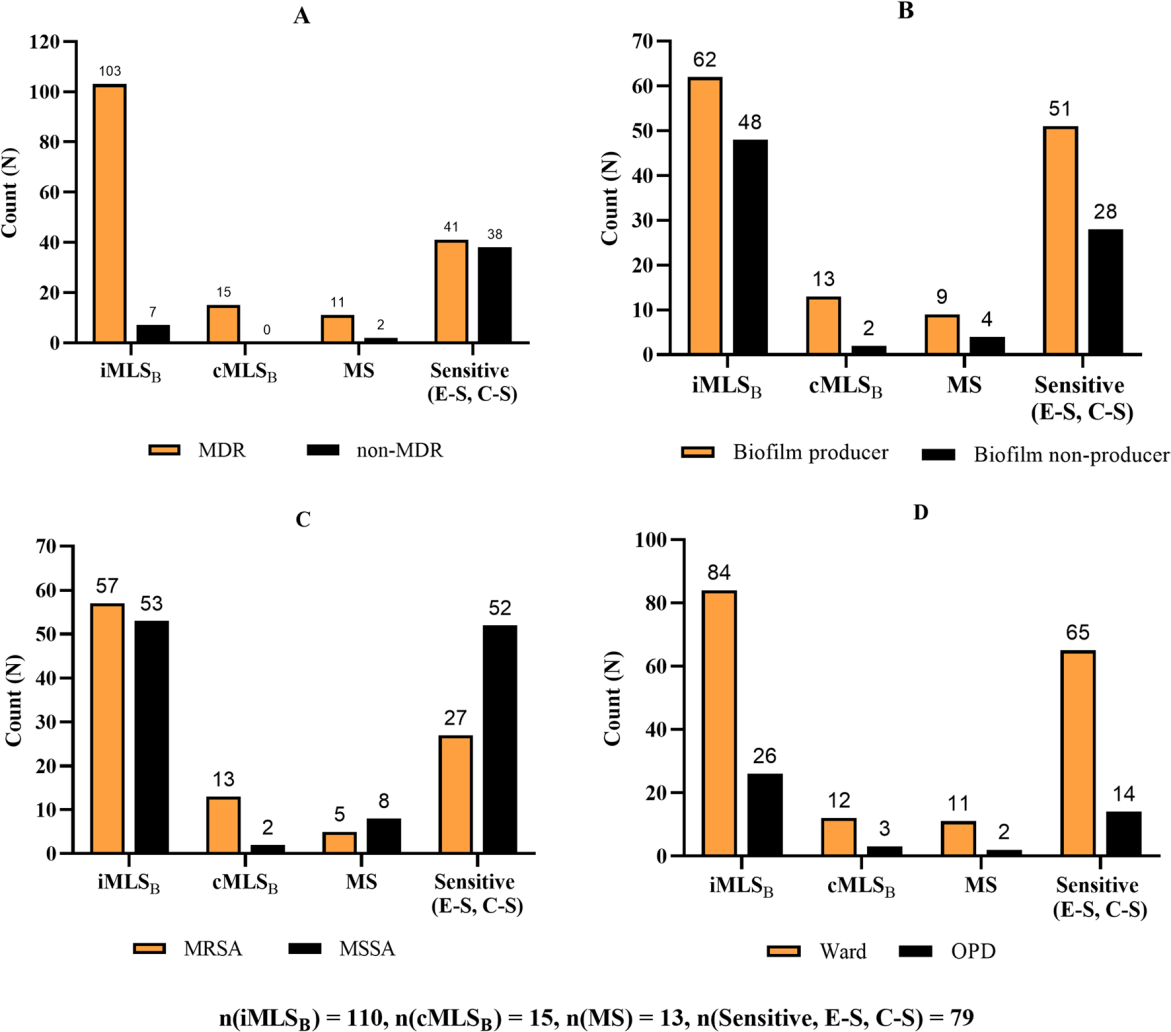
Distribution of *S. aureus* phenotypes as identified by the D-test between: **A** MDR and non-MDR isolates, **B** biofilm producer and non-producers, **C** MRSA and MSSA isolates, **D** isolates from ward and OPD

Of the total 217 isolates, 79 (36.4%) were sensitive to both erythromycin and clindamycin, 110 (50.7%) iMLS_B_, 15 (6.9%) cMLS_B_ and 13 (6.0%) were MS-phenotype. Inducible clindamycin resistance was significantly higher among MDR isolates (*p* = 0.0001). iMLS_B_ and cMLS_B_ phenotypes were higher in MRSA, whereas MS-type and S-type were higher in MSSA isolates. Although the number of iMLS_B_ phenotypes was higher in biofilm producers and MRSA isolates than in their counterparts, the differences were not statistically significant; *p* = 0.0927 and *p* = 0.01743, respectively. Also, there was no significant difference in the distribution of the D-test phenotypes among the hospital units (Fig. 5).

### MIC level of vancomycin among MRSA

**Fig. 6:**
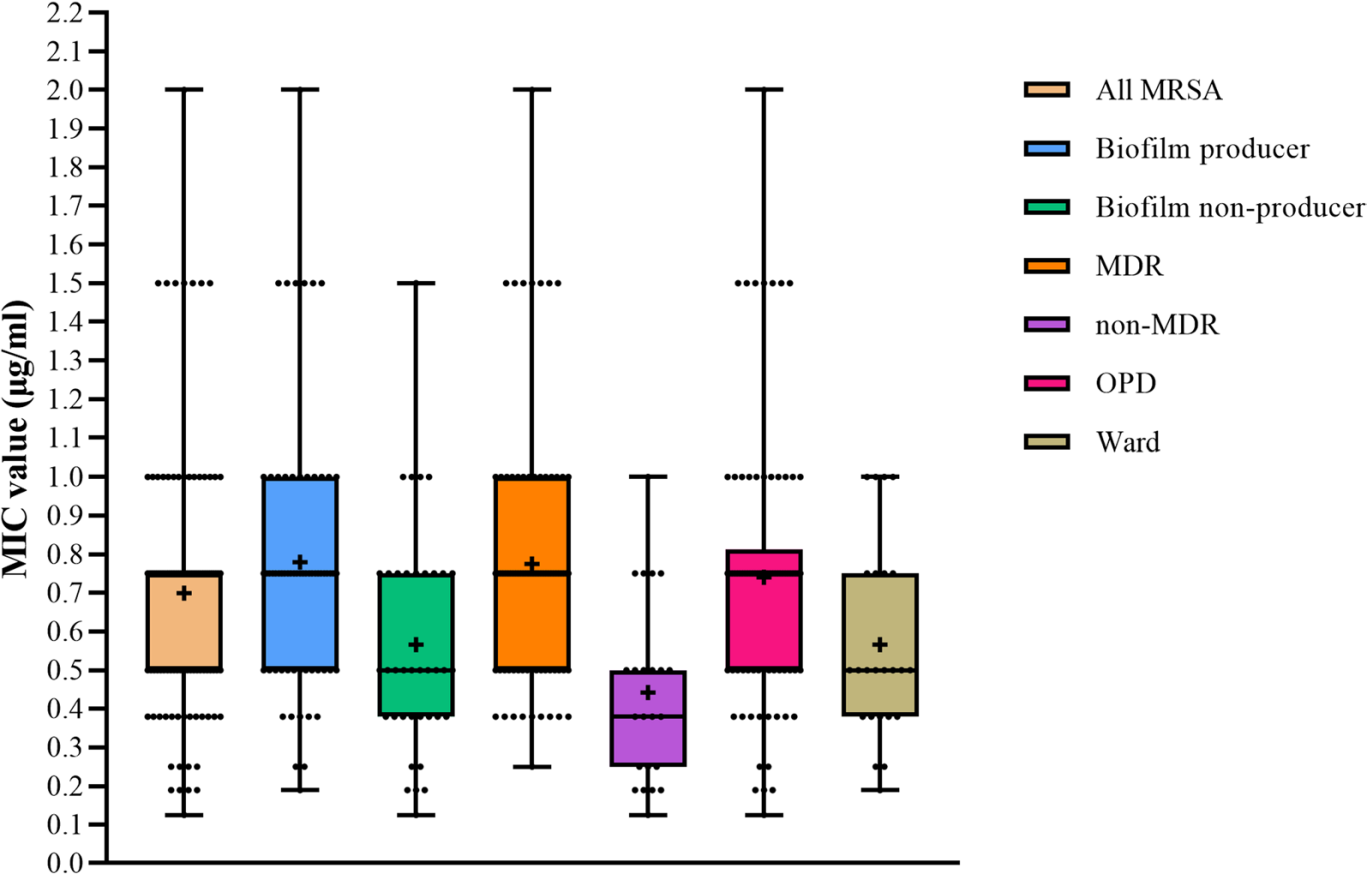
Distribution of MIC values of vancomycin of MRSA isolates

All MRSA isolates had MIC levels of vancomycin within the susceptible range (0.125–2 μg/ml) (Fig. 6).

The mean MIC of the MRSA isolates was 0.70 (*σ* = 0.35, SE = 0.034). Biofilm producers had a higher mean MIC value than biofilm non-producers with a significant difference in the means (*p* = 0.0013). MDR isolates had significantly higher mean values than the non-MDR isolates (*p* < 0.0001). Similarly, isolates from OPD also showed elevated MIC values than those from the ward (*p* = 0.0102) (Table 3).

**Table 3:** Mean MIC values of vancomycin of the isolates

	Mean (95% CI of mean)	S.D.	S.E. of mean	*p*-value
All MRSA	0.70 (0.63–0.77)	0.35	0.034	
Biofilm producer	0.78 (0.69–0.87)	0.35	0.044	0.00013^*^
Biofilm non-producer	0.57 (0.47–0.66)	0.29	0.047	
MDR	0.77 (0.70–0.85)	0.34	0.038	< 0.0001^*^
Non-MDR	0.44 (0.34–0.54)	0.23	0.049	
OPD	0.74 (0.66–0.82)	0.36	0.041	0.0102^*^
Ward	0.57 (0.46–0.67)	0.25	0.051	

^*^Indicates significant values

S.D.: standard deviation, S.E.: standard error

## Discussion

Due to the ability to produce many virulence factors, *S. aureus* is primarily responsible for skin and soft tissue infections in humans [21]. Although many studies have reported MRSA, very few reports on biofilm formation and the prevalence of clindamycin resistance among S. *aureus* have been published in the scenario of Nepal [11, 22, 23]. The information on the characterization and antibiotic susceptibility pattern of clinical *S. aureus* isolates would help a clinician evaluate its virulence and devise an appropriate treatment plan to manage staphylococcal infections.

Nearly half (47.0%) of the *S. aureus* isolates were MRSA in this study. Likewise, 78.3% of the isolates were MDR. A high MDR in *S. aureus*, was reported by Bhatta et al. [24] which is as consistent with our findings [24]. A similar prevalence of MRSA and MSSA was also reported by Lall and Sahni [25]. A higher prevalence of MSSA was also reported by Prabhu et al. [11] and Adhikari et al. [26]; however, the difference was relatively a little higher than in this study. In contrast to our findings, Majhi et al. [27] reported a higher prevalence of MRSA. These differences among studies could be attributed to variations in infection control practices and antibiotic prescription patterns in different settings. There was a significant association between multidrug resistance and methicillin resistance. A similar association was reported by Bhatta et al. [24].

In this study, 62.2% of *S. aureus* were biofilm producers, which agreed with Iorio et al. [28]. However, some other authors have reported a lower prevalence of biofilm-producing *S. aureus* [22, 29, 30]. A majority of the isolates were weak biofilm producers, comparable to Rezaei et al. [31]. This difference in the prevalence of biofilm-producing *S. aureus* isolates might be because the capability of *S. aureus* clinical isolates to form a biofilm is strain-specific and associated with different environmental conditions, which in turn depends on the differences in biofilm-related genes, genetic makeup, and physiological situation [32]. MRSA isolates usually have a higher biofilm-producing ability as the mecA gene of MRSA encodes penicillin-binding protein (PBP2a) and inactivates the accessory gene regulator (agr) quorum-sensing regulator system, which enhances biofilm formation [33]. The biofilm formation of MRSA is also enhanced by a phenol-soluble modulin mec (PSMmec) encoded by psm-mec [34]. However, in this study, both MRSA and MSSA showed an identical capacity to form substantial biofilm structures on the polystyrene pegs suggesting no correlation between methicillin susceptibility and biofilm formation (*p* = 0.879). A similar finding was reported by Smith et al. [35], while O'Neill et al. [36] reported a higher rate of biofilm formation in MSSA than in MRSA isolates. In contrast to this study, a higher ability of MRSA to form biofilm was reported by Mirani et al. [37] and Moghadam et al. [38].

A high level of sensitivity of clindamycin, amikacin, imipenem, and levofloxacin was observed in this study. None of the isolates were resistant to teicoplanin, chloramphenicol, doxycycline, and vancomycin. Similarly, common antibiotics like amoxycillin, erythromycin, piperacillin, and ciprofloxacin showed poor efficacy. A similar resistance pattern was shown in a study done by Iileka et al. [39] and Cp et al. [40]. The higher prevalence of drug-resistant isolates to such antibiotics could be associated with abuse of these drugs, poor hospital attendance, lack of public awareness, and the need for a better enlightenment campaign against the use of the drug without prescription [41].

We observed an increased resistance to available antibiotics in MRSA than in MSSA, consistent with previous studies [24, 42]. Methicillin resistance was significantly associated with multidrug resistance. All the MRSA isolates were resistant to amoxycillin, cephalexin, cefixime, and cloxacillin. On the positive side, teicoplanin, doxycycline, chloramphenicol, and vancomycin showed 100% sensitivity regardless of methicillin sensitivity and biofilm-producing ability. The efficiency of erythromycin and ciprofloxacin against the MRSA isolates was also abysmal. Higher resistance to erythromycin and clindamycin in MRSA isolates than MSSA isolates was also reported by Adhikari et al. [26]. These results emphasize the requirement of long-term surveillance and monitoring of antimicrobial susceptibility patterns of MRSA. This trend of emergence of antimicrobial resistance should be interrupted by developing an appropriate, rational, and prudent parent standard for drugs and implementing proper stewardship programs in the health care center and hospitals to protect antibiotics for future generations.

We did not observe any association between biofilm formation and MDR phenotype, which disagrees with other studies [43, 44]. Contrary to our findings, Song et al. [45] showed that biofilm producers of *S. aureus* have a greater likelihood of carrying more antibiotic resistance genes than biofilm non-producers. Biofilm producers showed higher resistance to available antibiotics, mainly amoxicillin and erythromycin. Furthermore, the biofilm producers had elevated resistance to most antibiotics compared to the non-producers. This finding was similar to the findings of Hassan et al. [30] and Sanchez et al. [46]. Rezaei et al. [31] showed higher resistance to erythromycin, clindamycin, and ciprofloxacin in biofilm producers than ours.

In this study, inducible clindamycin resistance (iMLS_B_) was observed in 50.7% of the total isolates, and a much lower prevalence of MS (6.0%) and cMLS_B_ (6.9%) phenotypes was observed. The prevalence of iMLS_B_ in this study is higher than previous findings in similar settings [1, 11, 44]. The prevalence of MS type and cMLS_B_ was higher in those studies except in Regmi et al. [47], where cMLS_B_ was lower than ours. In this study, iMLS_B_ was significantly higher in MDR isolates (*p* = 0.0001). Similarly, both iMLS_B_ and cMLS_B_ phenotypes were predominant in MRSA isolates, which is in close agreement with Adhikari et al. [1]. Although biofilm producers and MRSA isolates had higher iMLS_B_, no significant association was seen between them. Some other authors also reported higher iMLS_B_ phenotypes among MRSA isolates [11, 25]. Unlike ours, Eksi et al. [48] reported higher iMLS_B_ in MSSA than MRSA, however, with an insignificant difference like ours. Clindamycin is a reserve drug and is usually advocated in severe MRSA infections depending on the antimicrobial susceptibility results [11]. However, the possibility of the emergence of clindamycin resistance during therapy has raised concern over clindamycin prescription. Although the primary AST showed the maximum efficiency of clindamycin (93.1%), D-test detected inducible clindamycin resistance in more than half (54.5%) of the clindamycin sensitive isolates. Thus, D-test should be mandatory before clindamycin prescription to avoid clindamycin treatment failure.

Antibiotic treatment failure and the low success rate of treating MRSA infections with vancomycin are major public health concerns. The occurrence of VRSA has left us with limited antibiotics available for its treatment and is emerging as a severe public health problem [49]. In this study, no VRSA was detected, and all MRSA had MIC of vancomycin within the susceptible range (0.125–2 μg/ml); however, many of them had the MIC level near the upper limit of the CLSI susceptible range. This is consistent with other studies [23, 26, 50]. The increasing MIC level known as MIC creep has also been reported from many places around the globe [8, 51]. Moreover, biofilm production showed a strong statistical association with the MIC level of vancomycin (*p* = 0.0013), which agrees with Antunes et al. [52]. Higher MIC level of vancomycin in biofilm-producing isolates is mainly due to the enhanced potential for transfer of antimicrobial resistance genes, including resistance to vancomycin in the microenvironment of the biofilm [53]. Likewise, the potential for interspecies transfer of antimicrobial resistance genes, including resistance to vancomycin, may be enhanced by the microenvironment of a biofilm [53].

The increase in antibiotic resistance in *S. aureus*, the effect of biofilm on antibiotic resistance, shifting of MIC of vancomycin to higher levels, and increasing prevalence of iMLS_B_ phenotypes raise questions on the continued utility of the commonly used antibiotics, mainly erythromycin, clindamycin, and vancomycin to treat Staphylococcal infections and calls for prompt preventive actions [50]. The condition is even poorer in the poor and developing countries and can mainly be attributed to the widespread practice of empirical therapy, easy availability of drugs over the counter, self-medication practice, and inadequate infection-prevention practices in hospitals [41]. Hence, to prevent this situation of drug resistance from worsening, judicious use of these antibiotics based on culture and sensitivity reports should be promoted and new therapeutic options for the treatment of such infections should be explored to be prepared in advance for the worse to come [50]. As drug resistance among bacterial pathogens is an evolving process routine surveillance and monitoring studies should be conducted to provide physicians with knowledge on the updated and most effective treatment of staphylococcal infections.

Although some resistance is inevitable to antibiotics, steps can be taken to curtail practices that cause and propagate resistance. Drugs like vancomycin should only be used as the last option, and combination therapy or alternative treatments should be explored to optimize outcomes in staphylococcal infections and prevent the resistance of such antibiotics. In this way, we might be able to maintain or prolong the efficacy of existing drugs. The findings of this study will be helpful in the formation of effective diagnostic approaches and stewardship policy of antimicrobial therapy for the treatment of staphylococcal infections in similar hospital settings.

## Conclusion

The high rate of MDR, MRSA, and biofilm formation detected in *S. aureus* highlights the gravity of antibiotic resistance in Nepal. The absence of VRSA in this study supports the use of vancomycin in the treatment of serious staphylococcal infections, however, only after evaluating its MIC, mainly in biofilm-forming isolates. Clindamycin should be prescribed only after performing the D-test to reduce clindamycin treatment failure. Other alternatives like teicoplanin, chloramphenicol, doxycycline, amikacin, and levofloxacin can still treat MRSA infections; however, it should be based on culture and sensitivity reports.

## Limitations of the study

A comparatively small size and shorter duration of this study might limit the generalization of this study. Furthermore, the study was limited to phenotypic detection. The study being conducted in a single hospital might also be a limitation. However, being a national-level hospital and the similar patient distribution and medical practices in almost all other hospitals, we believe our findings represent the scenario of the whole region. Also, only phenotypic characterization was performed.

## Supplementary Information

**Additional file 1: Photograph S1.** (A) Isolation of *S. aureus* in blood agar, (B) Identification of *S. aureus* using DNase media. *S. aureus* showing a clear zone around the colonies, (C) Antibiotic sensitivity test of *S. aureus*, (D) D-test: Flattening of Clindamycin adjacent to Erythromycin at a distance of 15 mm shows ICR. (E) Biofilm detection by Tissue culture plate method: Microtitre plate showing different levels of biofilm production, (F) Determination of MIC of Vancomycin by E-test method.

## Data Availability

The data supporting the conclusions of this article are included within the article.
