# Supplementary figures and images for: Phenotypic detection of methicillin resistance, biofilm production, and inducible clindamycin resistance in Staphylococcus aureus clinical isolates in Kathmandu, Nepal
Source: Trop Med Health. 2022 Sep 21;50:71. doi: 10.1186/s41182-022-00460-1 (PMC9490977; doi:10.1186/s41182-022-00460-1)

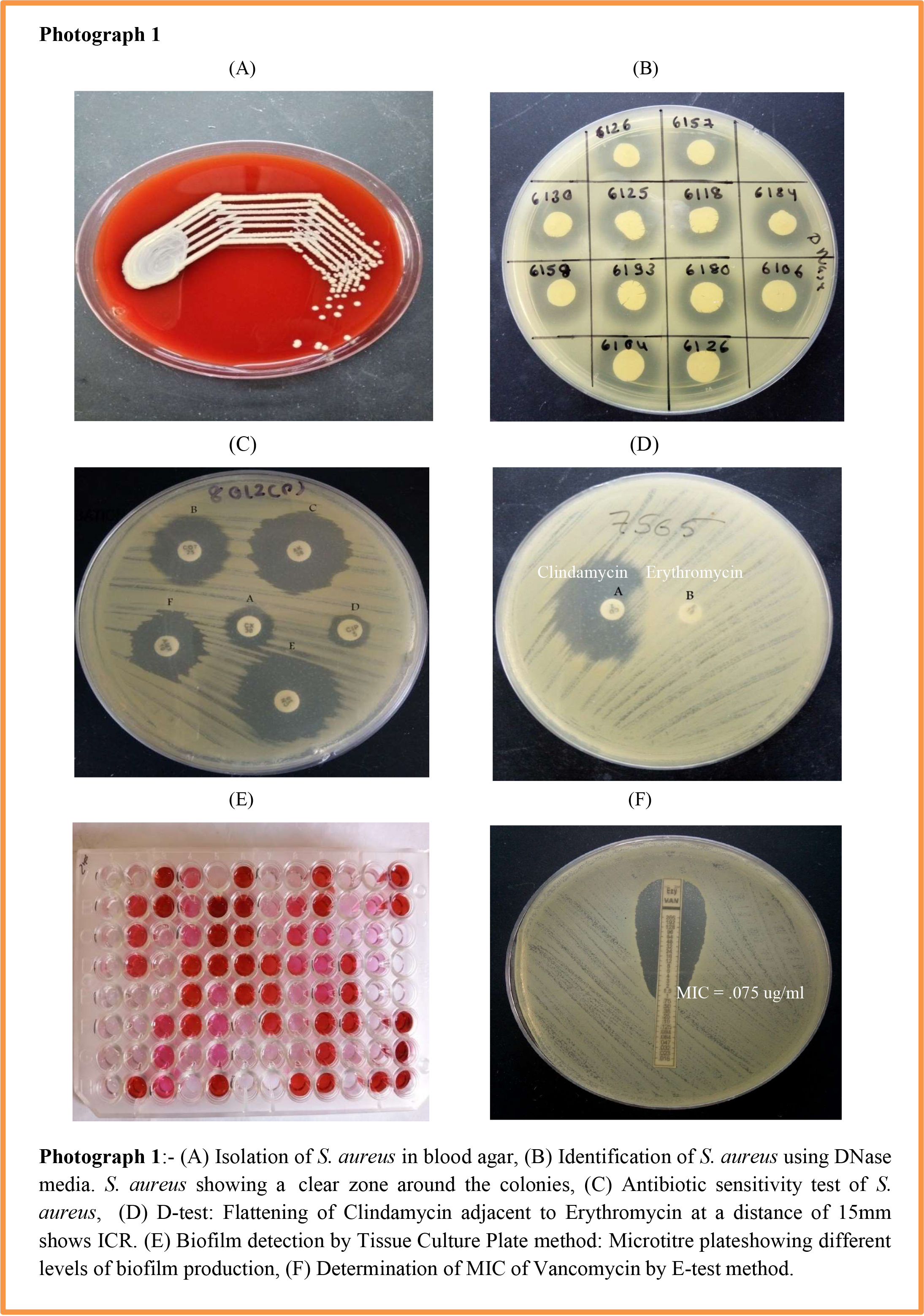

Supplement: Supplementary file 1 — Additional file 1: Photograph S1. (A) Isolation of S. aureus in blood agar, (B) Identification of S. aureus using DNase media. S. aureus showing a clear zone around the colonies, (C) Antibiotic sensitivity test of S. aureus, (D) D-test: Flattening of Clindamycin adjacent to Erythromycin at a distance of 15 mm shows ICR. (E) Biofilm detection by Tissue culture plate method: Microtitre plate showing different levels of biofilm production, (F) Determination of MIC of Vancomycin by E-test method. [file 41182_2022_460_MOESM1_ESM.jpg]
